# Draft genome sequence of *Nitrosomonas* sp. ANs5, an extremely alkali-tolerant ammonia-oxidizing bacterium isolated from Mongolian soda lakes

**DOI:** 10.1128/mra.00330-25

**Published:** 2025-08-15

**Authors:** Jose R. Valera, Dimitry Y. Sorokin, Alyson E. Santoro

**Affiliations:** 1 Department of Ecology, Evolution, & Marine Biology, University of California, Santa Barbara, California, USA; 2 Research Centre of Biotechnology, Winogradsky Institute of Microbiology, Russian Academy of Sciences 230762 , Moscow, Russia; 3 Department of Biotechnology, TU Delft, Delft, the Netherlands; University of Maryland School of Medicine, Baltimore, Maryland, USA

**Keywords:** nitrification, extremophiles

## Abstract

The draft genome of a chemolithoautotrophic ammonia-oxidizing bacterium of the genus *Nitrosomonas* is reported. *Nitrosomonas* sp. strain ANs5, previously classified as a strain of *N. halophila*, is an alkali-tolerant ammonia-oxidizing bacterium isolated from the soda lakes of northeast Mongolia.

## ANNOUNCEMENT

*Nitrosomonas sp*. strain ANs5 (hereafter ANs5) is one of five nitrosomonads isolated from composite sediment samples of the saline soda lakes in the northeast region of Mongolia (Choibolsan Province) (1). ANs5 is a halotolerant, ammonia-oxidizing bacterium (AOB) in the order of *Burkholderiales*, in the family of *Nitrosomonadaceae* (Betaproteobacteria) that is capable of growth at pH values as high as 11.4 (1), the highest pH known for any bacterium in the *Nitrosomonadaceae*. ANs5 was originally classified as a strain of *Nitrosomonas halophila*, of which the type strain is strain Nm1, and expanded the species description to include alkali-tolerant properties (2). Genome comparisons between ANs5, Nm1, and non-alkaliphilic strains may lead to better understanding of alkaliphilic adaptations within the AOB.

ANs5 was grown in batch culture on a temperature-controlled shaker set to 100 rpm at 30°C in 160 mL glass screw-top bottles containing 30 mL of media. The alkaline (pH = 9.7–10) medium was prepared as previously described, with 10 mM ammonium (1). Eight cultures were combined and collected via vacuum filtration on a 25 mm, 0.22 µm pore-sized polyethersulfone (Pall Supor) membrane filter. DNA was extracted using a modification of the DNeasy Blood & Tissue kit (Qiagen) as previously described (3). DNA libraries were constructed using the Illumina DNA Prep kit and sequenced using the Illumina NovaSeq X Plus platform with 2 × 151 bp paired-end reads, producing a total of 1,541,784 paired reads.

**Fig 1:**
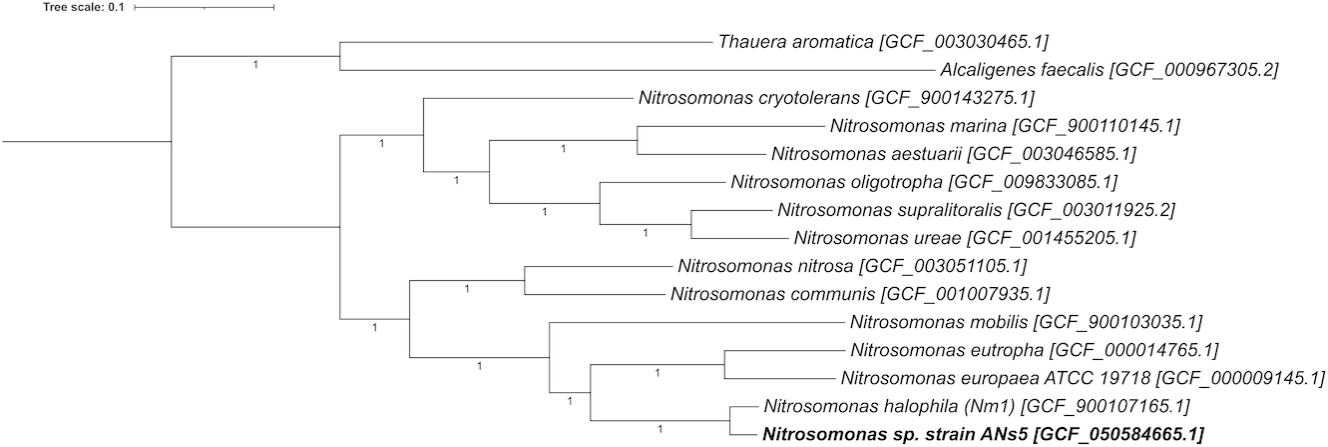
A maximum-likelihood phylogenetic tree of representative ammonia-oxidizing bacteria genomes visualized with iTOL (v.7.2) (13). The UFBoot support values are indicated below branches. The phylogenetic tree is rooted, with the outgroup represented by betaproteobacteria outside of the genus *Nitrosomonas*.

The draft genome was analyzed using the Kbase open-source platform for genome assembly and analysis (4). Default parameters were used except where otherwise noted. Quality control and preprocessing were conducted with FASTQC (v.0.12.1) (5), TRIMMomatic (v.0.36; sliding window=5, min. quality=20) (6), and PRINSeq (v.0.20.4) (7), in that order. Processed reads were assembled using Unicycler (v.0.4.8; min. contig length =2000 bp, contig bridging threshold = ‘bold’) (8). The assembled genome (read coverage = 65×) was annotated via NCBI PGAP (v 6.8) (9). Our phylogenetic analysis, based on a concatenated alignment of single-copy genes in the Proteobacteria HMM set (*n*=119 genes) within GToTree (v.1.8.10) (10) was used to construct the maximum-likelihood tree with FastTree2 (v.2.1.11) (11), visualized with iToL (v.7.2) (12) (Fig. 1).

The final assembly contains 122 contigs with an N_50_ value of 49,119, totaling 3.07 Mbp, and a GC content of 52% (Table 1). The genome contained 2,713 protein-coding genes and is 99.82% complete with 0.42% contamination, estimated by CheckM2 (v.1.1.0) (13). Genes encoding chemolithotrophic ammonia oxidation were identified, including two copies of the ammonia monooxygenase subunits (*amoCAB;* ABTW62_11265:75 & ABTW62_07675:85), and a single copy of the hydroxylamine oxidoreductase (*hao*; ABTW62_13545) gene. We identified the gene encoding nitrite reductase (*nirK*; ABTW62_02095). Strains ANs5 and Nm1 share an average nucleotide identity (ANI) of 91.9% as determined by FASTANI (v.0.1.3) (14). Using the Compare Two Proteomes tool in KBase with a <60% amino acid identity threshold for unique genes, we identified 500 genes unique to ANs5 that are not shared with Nm1.

**TABLE 1:** Genome features of *Nitrosomonas* sp. strain ANs5

Strain	NCBI accession number	Genome size (bp)	Gc (%)	No. of contigs	No. of total genes	No. of protein CDS	No. of rRNA operons	No. of tRNA operons	Completeness / Contamination (%)
*Nitrosomonas* sp. ANs5	PRJNA1125436	3,066,906	52.34	122	2,782	2,713	2	38	99.82/0.42

## Data Availability

The genome assembly is deposited at DDBJ/ENA/GenBank under BioProject PRJNA1125436. The raw sequencing data were deposited at NCBI SRA under accession number SRR29456553 and the assembly under accession number GCA_050584665.1.
